# Potential association between frailty and pTau in community-dwelling older adults

**DOI:** 10.1186/s12877-022-03454-0

**Published:** 2022-09-26

**Authors:** Lixing Zhou, Hui Shi, Rui Cheng, Meiling Ge, Fengjuan Hu, Lisha Hou, Xin Xia, Xiaolei Liu, Yixin Liu, Yunli Zhao, Linghui Deng, Wanyu Zhao, Zhiliang Zuo, Xuelian Sun, Jirong Yue, Birong Dong

**Affiliations:** 1 National Clinical Research Center for Geriatrics and Department of Geriatrics, West China Hospital, Sichuan University, No. 37 Guo Xue Alley, Chengdu, 610041 Sichuan China; 2 Department of Epidemiology, University of Pittsburgh, Pittsburgh, PA 15260 USA

**Keywords:** Frailty, Tau, Biomarker, Cognition

## Abstract

**Background:**

Frailty is a geriatric syndrome characterized by a decline in physiological reserves, and multiple factors contribute to the occurrence and development of frailty. Growing evidence supports a strong link and overlap between frailty and cognitive impairment, but the mechanisms involved have not yet been fully elucidated.

**Aim:**

To identify associations between 12 plasma cognition-related biomarkers and frailty in community-dwelling older adults.

**Methods:**

A total of 375 participants (age 70.9 ± 5.8, 165 men and 210 women) were included in this study. Frailty was assessed using the modified Fried frailty phenotype. Participants were divided into not-frail group (*n* = 313) and frail group (*n* = 62). Twelve plasma cognitive biomarkers were detected by enzyme-linked immunosorbent assay (ELISA). Multinomial logistic regression was used to explore the association between different biomarkers and frailty status.

**Results:**

Among the 12 biomarkers, only pTau was higher in frail individuals than in their not-frail peers (471.3 ± 58.1 pg/mL vs. 451.9 ± 61.1 pg/mL, *p* = 0.022). No other biomarkers had any significant association with frailty, including total-Tau (tTau), neurofilament light (NFL), amyloid-β 40 (Aβ40), amyloid-β 40 (Aβ42), S100 calcium binding protein B (S100B), visinin-like protein 1 (VLP-1), Alzheimer-associated neuronal thread protein (AD7cNTP), β-amyloid precursor protein (βAPP), chitinase-3-like-1 (CHI3L1), soluble complement receptor 1 (sCR1) and heart-type fatty acid binding protein (hFABP). Furthermore, pTau was compared between negative and positive subject groups for each individual criterion of frailty. Significantly higher levels of pTau were observed in those who were positive for the criteria of low grip strength (451.2 ± 61.4 pg/mL vs. 469.1 ± 57.6 pg/mL, *p* = 0.019), exhaustion (451.2 ± 61.6 pg/mL vs. 466.4 ± 58.4 pg/mL, *p* = 0.035) and low physical activity (451.1 ± 60.7 pg/mL vs. 465.7 ± 60.7 pg/mL, *p* = 0.034) when compared to those who were negative for each corresponding criterion. Finally, in the multivariable-adjusted analysis, the association between pTau and frailty was statistically significantly associated (OR: 1.40, 95% CI: 1.04–1.89), even after adjusting.

**Conclusions:**

The present study found a potential association between pTau and frailty. Future works should monitor the longitudinal trajectory of changes of pTau concentrations in frailty older adults. A better understanding of the molecular mechanisms behind will contribute to biomarker research in frailty.

## Introduction

Frailty is a geriatric syndrome characterized by a decline in physiological reserves, which increases one’s vulnerability to endogenous or exogenous stressors and is associated with poorer quality of life among many adverse outcomes [1–5]. Among older adults, frailty is associated with an increased risk of adverse health outcomes (including disease incidence, hospitalization, institutionalization, mortality, etc.), no matter what physical measurements were used through the frail phenotype or multidimensional assessment by the frailty index [6–8]. The reason why frailty has attracted much attention in recent years is because it facilitates the identification of a subgroup of older adults who are at high risk of adverse health outcomes, including institutionalization, recurrent hospitalization and premature death [9], and it represents an early step into disability, which may be reversible [5, 10, 11].

Because there is still no gold standard for the clinical diagnosis of frailty, one of the current research priorities is to find some plasma biomarkers of frailty to help diagnosis, which may help to elucidate the mechanism of frailty. However, to date, studies investigating the relationship between biomarkers and frailty have mainly focused on inflammatory biomarkers, and the conclusions are inconclusive. While some studies have shown that elevated inflammatory biomarkers are correlated with frailty [12, 13], others have shown no association between higher inflammatory biomarker levels and the incidence of frailty [14–16]. Therefore, these biomarkers are not necessarily helpful in the diagnosis of frailty.

To date, numerous cross-sectional and longitudinal studies have shown a significant association between physical frailty and cognitive impairment or dementia [17–21]. Older adults with cognitive declines usually demonstrate decreased physical performance. A growing number of evidence indicates that people without frailty are better able to resist neurodegeneration [21–25], whereas those with worse frailty status are more likely to have Alzheimer's disease (AD) and dementia [21]. Frail older adults are at higher risk of cognitive decline, which inversely increases the probability of incident of frailty [20]. This suggests that the co-occurrence of each disease has an effect on the other [26, 27], and the etiology of these two diseases may be correlated. However, the mechanisms involved in this relationship have not yet been fully elucidated.

Given that it is likely that frailty and cognition share some common risk factors and biological mechanisms [28], evaluating the associations between plasma cognitive biomarkers and frailty may help to better understand the biological mechanisms behind and to identify new biomarkers of frailty. Since multiple molecular pathways are involved in the neurodegenerative process and all may contribute to various aspects of frailty, we measured a panel of 12 different cognitive biomarkers according to literatures [29–34], including total-Tau (tTau), phospho-Tau (pTau, Thr181), neurofilament light (NFL), amyloid-β 40 (Aβ40), amyloid-β 40 (Aβ42), S100 calcium binding protein B (S100B), visinin-like protein 1 (VLP-1), Alzheimer-associated neuronal thread protein (AD7cNTP), β-amyloid precursor protein (βAPP), chitinase-3-like-1 (CHI3L1, also termed YKL-40), soluble complement receptor 1 (sCR1) and heart-type fatty acid binding protein (hFABP).

## Methods

### Study population

The study samples were obtained from the baseline of the West China Health and Aging Trend (WCHAT) study. The study was approved by the Ethics Committee of West China Hospital, Sichuan University (No. 2017–445) and registered in the Chinese Clinical Trial Registry (ChiCTR1800018895). This is an ongoing, prospective cohort study starting in 2018 to assess the health status and its influencing factors in Western China. The baseline survey of the WCHAT study included 7536 people aged 50 or over from 18 ethnic groups in four provinces. According to the Declaration of Helsinki Ethical Principles, all participants signed a written informed consent form to participate in the trial. Participant information was collected through face-to-face interviews [35].

Among the WCHAT cohort, 378 participants had both their cognition-related biomarkers data and frailty data. After excluding 3 individuals with AD or other psychiatric disorders, we finally included 375 participants in the current analysis.

### Blood sample collection and enzyme-linked immunosorbent assay (ELISA)

Fasting peripheral blood was collected by a trained nurse when participants arrived at the research center in the morning. Routine blood tests and biochemical parameters were detected by a chemistry analyzer (Olympus AU400, Tokyo, Japan) and a hematology analyzer (MEDONIC CA620, Spånga, Sweden), respectively. Other blood samples were centrifuged at 3500 × g for 15 min within 30 min after venipuncture. Plasma was collected and stored at -80 ℃. Blood handling procedures were performed under a strict standardized protocol.

Cytokines and biomarkers in plasma were measured using a commercially available ELISA kit (eBioscience, San Diego, CA, USA) according to the manufacturer’s protocols.

### Assessment of frailty

Frailty was assessed using the modified Fried frailty phenotype [10], consisting of the following five criteria: weakness, shrinking, slowness, exhaustion and inactivity. As described previously [35], participants meeting three or more criteria were classified as frail, and those meeting two or fewer criteria were categorized as not-frail.

- 1 Weakness: weakness was defined using maximum grip strength of the dominant hand as ≤ 20th percentile of the population distribution, adjusted for sex and body mass index (BMI).
- 2 Shrinking: shrinking was ascertained by loss of weight for more than 4.5 kg during the last year or having a BMI < 18.5 kg/m2.
- 3 Slowness: slowness was defined using the average of the timed walk test over a 4-m course as the ≤ 20th percentile of the population distribution, adjusted for sex and standing height.
- 4 Exhaustion: meeting one of the following three criteria was considered exhaustion. (1) I felt excessively fatigued most of the time; (2) I felt excessively weak most of the time; (3) The self-reported energy score was no more than 3, when 10 represents the most powerful condition.
- 5 Inactivity: the bottom quintile of sex-adjusted kilocalories (kcals) from a validated China Leisure Time Physical Activity Questionnaire (CLTPAQ) [36]. The CLTPAQ is a modified version of the Minnesota Leisure Time Physical Activity Questionnaire (MLTPAQ) [37] based on the Chinese lifestyle and cultural background.

### Covariates

The analyses were adjusted for several demographic variables, health-related and functional variables, and clinical risk factors. Demographic factors included age, sex, education (illiterate, primary school, secondary school and above), ethnicity (Han, Tibetan, Yi, Uighur and others) and marital status (married, and single (unmarried/widowed/divorced)). Health-related and functional variables included history of smoking, disability in activities of daily living (ADL disability), falls in the last year, number of chronic conditions and depression. ADL disability was defined as having the need for assistance or difficulty in one or more of the ten items in the Barthel Index. Fall status in the last year was dichotomized as having had falls versus having no falls in the last two years. The number of chronic diseases was categorized as 0, > 1 and ≥ 2 based on the doctor's diagnosis of hypertension, heart disease, lung disease, digestive disease, stroke, diabetes, osteoarthritis, and tumor. Depression was evaluated by the 15-item Geriatric Depression Scale (GDS-15). Individuals with a GDS-15 score of 5 or greater were classified as having depression. Body mass index (BMI) was calculated as weight (kilograms) divided by height (meters) squared and then classified into nonobese (BMI < 30.0 kg/m^2^) and obese (BMI ≥ 30.0 kg/m^2^) groups. Nutritional status was assessed using the Mini Nutritional Assessment (MNA-SF). If the MNA-SF score was ≤ 12, the subjects were defined as malnourished. Cognitive function was evaluated by the Short Portable Mental Status Questionnaire (SPMSQ), with a score ranging from 0–10 and a higher score representing a poor cognitive function. A score of more than 4 in individuals with primary school education and less or a score of more than 2 in individuals with high school education and higher are defined as cognitive impairment.

### Statistical analysis

Analysis of variance (ANOVA) and chi-square tests were used to compare the distributions of continuous and categorical variables, respectively, by frailty status. To examine the association between frailty and biomarkers, we used generalized linear models (GLM) to fit three models as follows: (1) unadjusted model 1 (crude model); (2) model 2 adjusted by age and sex; and (3) model 3 adjusted by the variables in model 2 plus education, marital status, comorbidity, depression, cognition, and obesity. The results were reported per 1-SD increase in circulating concentrations.

A *p* value < 0.05 was considered statistically significant, and all analyses were performed using Stata statistical software version 15.1 (Stata Corp, College Station, TX), R statistical software version 4.0.3 (in R Studio 1.4.1106 environment) and GraphPad Prism version 8 (GraphPad Software, San Diego, CA).

## Results

### Characteristics of the study sample by frailty status

The characteristics of the study participants by frailty status are presented in Table 1. Overall, we included 375 participants (165 men and 210 women) in this study. The mean age was 70.9 ± 5.8 years. Participants were defined as not-frail (*n* = 313) and frail (*n* = 62) by the modified Fried frailty phenotype. Compared to not-frail group, the frail group had a higher percentage of Uighur ethnicity, comorbidity, single status, activities of daily living (ADL) disability, risk of malnutrition and cognitive function impairment. Interestingly, the obesity rate was higher in the frail group.

**Table 1:** Characteristics of the study sample by frailty status (*n* = 375)

		Frailty status		
	Overall, *N* = 375 ^a^	Not-frail, *N* = 313 ^a^	Frail, *N* = 62 ^a^	*p* value ^b^
**Age (years), mean (SD)**	70.9 (5.8)	70.9 (5.7)	71.3 (6.5)	0.612
**Age (years), n (%)**				0.172
60–69	140 (37%)	114 (36%)	26 (42%)	
70–79	204 (54%)	176 (56%)	28 (45%)	
≥ 80	31 (8.3%)	23 (7.3%)	8 (13%)	
**Sex, n (%)**				0.937
Men	165 (44%)	138 (44%)	27 (44%)	
Women	210 (56%)	175 (56%)	35 (56%)	
**Education background, n (%)**				0.077
Illiterate	125 (33%)	112 (36%)	13 (21%)	
Primary school	116 (31%)	93 (30%)	23 (37%)	
Secondary school and above	134 (36%)	108 (35%)	26 (42%)	
**Ethnicity, n (%)**				< 0.001
Han	171 (46%)	158 (50%)	13 (21%)	
Tibetan	22 (5.9%)	21 (6.7%)	1 (1.6%)	
Yi	42 (11%)	40 (13%)	2 (3.2%)	
Uighur	118 (31%)	76 (24%)	42 (68%)	
Others	22 (5.9%)	18 (5.8%)	4 (6.5%)	
**Marital status, n (%)**				0.017
Married	265 (71%)	229 (73%)	36 (58%)	
Unmarried/widowed/divorced	110 (29%)	84 (27%)	26 (42%)	
**Number of chronic conditions, n (%)**				< 0.001
0	214 (57%)	192 (61%)	22 (35%)	
1	91 (24%)	79 (25%)	12 (19%)	
≥ 2	70 (19%)	42 (13%)	28 (45%)	
**Falls in last year, n (%)**	63 (18%)	48 (16%)	15 (25%)	0.096
**History of smoke, n (%)**	97 (26%)	76 (24%)	21 (34%)	0.115
**ADL disability, n (%)**	66 (18%)	37 (12%)	29 (47%)	< 0.001
**Depression, n (%)**	60 (16%)	46 (15%)	14 (23%)	0.122
**Obesity, n (%)**	47 (13%)	31 (9.9%)	16 (26%)	< 0.001
**Nutritional status, n (%)**				0.006
Normal	255 (68%)	222 (71%)	33 (53%)	
Risk of malnutrition	120 (32%)	91 (29%)	29 (47%)	
**Cognitive function impairment, n (%)**				0.001
Normal	296 (79%)	258 (82%)	38 (61%)	
Mild	55 (15%)	38 (12%)	17 (27%)	
Medium	22 (5.9%)	16 (5.1%)	6 (9.7%)	
Severe	2 (0.5%)	1 (0.3%)	1 (1.6%)	

^a^ Frequency (%)

^b^ Pearson's Chi-squared test; Fisher's exact test

### The level of plasma pTau was elevated in frail individuals

**Fig. 1:**
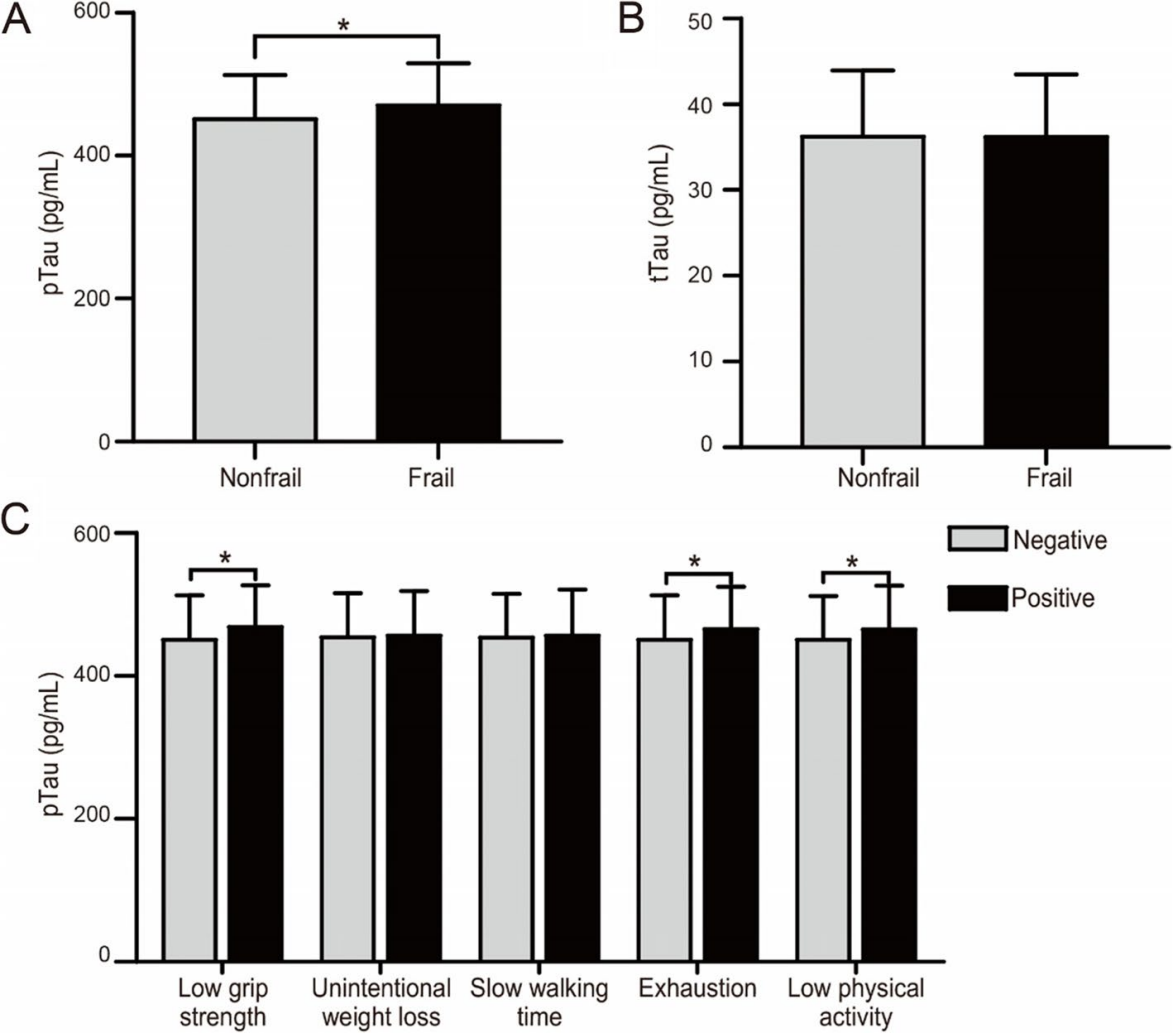
The levels of cognitive biomarkers in the study groups. PTau (**A**) and tTau (**B**) were detected and classified according to different frailty statuses. The level of pTau was analyzed in the population according to each frailty criterion (**C**). Data are expressed as the means ± SD. *, *p* < 0.05

According to references [29–34], 12 cognitive biomarkers in plasma were detected in our study, including tTau, pTau (Thr181), NFL, Aβ40, Aβ42, S100B 1, VLP-1, AD7cNTP, βAPP, CHI3L1, sCR1 and hFABP. The concentrations of each biomarker are described in Table 2. Among the 12 biomarkers, only pTau was significantly higher in frail individuals than in their not-frail peers (471.3 ± 58.1 pg/mL vs. 451.9 ± 61.1 pg/mL, *p* = 0.022, Fig. 1A). No other biomarkers had any significant association with frailty, including tTau (36.2 ± 7.2 pg/mL vs. 36.3 ± 7.7 pg/mL, *p* = 0.992, Table 2 and Fig. 1B).

**Table 2:** Cognition-related biomarkers by frailty status (*n* = 375)

		Frailty status		
	Overall, *N* = 375 ^a^	Not-frail, *N* = 313 ^a^	Frail, *N* = 62 ^a^	*p* value
**tTau**	36.3 (7.6)	36.3 (7.7)	36.2 (7.2)	0.992
**pTau**	455.1 (61.0)	451.9 (61.1)	471.3 (58.1)	0.022
**NFL**	616.1 (53.3)	617.1 (53.9)	610.6 (50.3)	0.378
**Aβ40**	62.1 (12.5)	62.1 (12.5)	62.2 (12.3)	0.931
**Aβ42**	317.3 (77.5)	316.7 (77.8)	320.4 (76.3)	0.732
**S100B**	654.8 (95.9)	654.1 (95.5)	658.3 (98.3)	0.756
**VLP-1**	219.4 (49.4)	217.4 (48.8)	229.2 (51.9)	0.088
**AD7cNTP**	1026.9 (379.4)	1016.4 (379.4)	1079.4 (378.2)	0.233
**βAPP**	1966.6 (467.4)	1977.5 (467.3)	1912.8 (468.2)	0.313
**CHI3L1**	60.1 (13.9)	60.1 (14.1)	60.3 (13.4)	0.905
**sCR1**	47.1 (6.2)	47.0 (6.2)	47.5 (6.2)	0.518
**hFABP**	10.7 (2.5)	10.7 (2.5)	10.9 (2.7)	0.622

^a^ Mean pg/mL (SD)

Given the positive influence of frailty on pTau assay results, to determine the single contribution of each frailty criterion to pTau levels, this parameter was compared between negative and positive subject groups for each individual criterion (Fig. 1C). No differences were identified between negative and positive individuals in terms of unintentional weight loss (454.7 ± 61.0 pg/mL vs. 457.1 ± 61.5 pg/mL, *p* = 0.767) or slow waking time (454.5 ± 60.4 pg/mL vs. 457.3 ± 63.5 pg/mL, *p* = 0.703). However, significantly higher levels of pTau were observed in those who were positive for the criteria low grip strength (451.2 ± 61.4 pg/mL vs. 469.1 ± 57.6 pg/mL, *p* = 0.019), exhaustion (451.2 ± 61.6 pg/mL vs. 466.4 ± 58.4 pg/mL, *p* = 0.035) and low physical activity (451.1 ± 60.7 pg/mL vs. 465.7 ± 60.7 pg/mL, *p* = 0.034) when compared to those who were negative for each corresponding criterion. The above results indicated that the level of pTau was elevated in frail individuals, where positivity for low grip strength, exhaustion and low physical activity may contribute.

### PTau was statistically significantly associated with frailty after multivariate logistic regression

In model 1, for each standard deviation (SD) increased in pTau, older adults with frailty had an odds ratio (OR) of 1.38 (95% confidence interval [CI]: 1.05–1.83) compared with not-frail adults. PTau remained statistically significantly associated after age and sex adjustment (OR: 1.41, 95% CI: 1.06–1.87) in model 2. Furthermore, in model 3, the association between pTau and incident frailty was still statistically significantly associated (OR: 1.40, 95% CI: 1.04–1.89). There were no statistically significant associations with other biomarkers between not-frail and frail patients. 

 (Table 3).

**Table 3:** Associations between cognition-related biomarkers and frailty (*n* = 375) ^a^

	Model 1 ^b^		Model 2 ^c^		Model 3 ^d^	
	OR (95% CI)	*p* Value	OR (95% CI)	*p* Value	OR (95% CI)	*p* Value
**tTau**	1.00 (0.76 to 1.31)	0.992	0.98 (0.75 to 1.30)	0.906	0.98 (0.72 to 1.33)	0.891
**pTau**	**1.38 (1.05 to 1.83)**	**0.023**	**1.41 (1.06 to 1.87)**	**0.019**	**1.40 (1.04 to 1.89)**	**0.029**
**NFL**	0.88 (0.67 to 1.16)	0.377	0.91 (0.69 to 1.21)	0.518	0.91 (0.67 to 1.23)	0.532
**Aβ40**	1.01 (0.77 to 1.33)	0.931	1.01 (0.77 to 1.33)	0.956	0.98 (0.73 to 1.31)	0.873
**Aβ42**	1.05 (0.80 to 1.38)	0.732	1.08 (0.82 to 1.43)	0.583	1.15 (0.85 to 1.56)	0.371
**S100B**	1.05 (0.80 to 1.37)	0.755	1.04 (0.79 to 1.36)	0.807	1.12 (0.83 to 1.52)	0.465
**VLP1**	1.27 (0.96 to 1.69)	0.089	1.28 (0.97 to 1.70)	0.086	1.15 (0.84 to 1.56)	0.384
**AD7cNTP**	1.18 (0.90 to 1.56)	0.233	1.18 (0.89 to 1.55)	0.246	1.29 (0.94 to 1.75)	0.112
**βAPP**	0.87 (0.66 to 1.14)	0.312	0.88 (0.67 to 1.16)	0.349	0.84 (0.62 to 1.13)	0.25
**CHI3L1**	1.02 (0.77 to 1.34)	0.905	1.00 (0.76 to 1.32)	0.978	1.08 (0.80 to 1.47)	0.61
**sCR1**	1.10 (0.83 to 1.44)	0.517	1.9 (0.83 to 1.45)	0.534	1.13 (0.83 to 1.54)	0.439
**hFABP**	1.07 (0.82 to 1.41)	0.621	1.08 (0.82 to 1.42)	0.601	1.08 (0.80 to 1.45)	0.636

^a^ For per 1-*SD* increase in cognition-related biomarkers

^b^ Unadjusted

^c^ Adjusted for age and sex

^d^ Adjusted for age, sex, education, marital status, comorbidity, depression, cognition and obesity

## Discussion

This study provides a comprehensive examination of the relationships between a wide range of plasma cognitive biomarkers and frailty in community-dwelling older adults, and is the first to find a potential association between plasma pTau levels and frailty in older adults.

To date, no particular biomarker has been consistently associated with frailty status. In the present study, we found that only pTau was elevated significantly in frail individuals, but none of the other measured plasma cognitive-related biomarkers were linked to frailty, suggesting that frailty is potentially associated with pTau. Only one study has examined tTau and pTau in frailty and found no significant relationship between them. However, they tested in cerebrospinal fluid while we used plasma [38]. Tau, a microtubule-associated protein, is abundant in neuronal axons and plays an important role in the assembly and stabilization of microtubules [39]. pTau is the active form of Tau. Recent studies indicate that cerebrospinal fluid Tau phosphorylated at position threonine 181 has diagnostic utility for several neurological disorders [40]. Although the major function of Tau in the brain remains to be determined, there is now much evidence implicating the protein Tau in the pathogenesis of a variety of cognition-related disorders, including AD and other neurodegenerative conditions [41]. The pathological hallmark of these diseases is the intraneuronal accumulation of insoluble filamentous Tau aggregates, leading to the formation of neurofibrillary tangles [39]. These functions may involve the regulation of signaling pathways associated with different biological processes.

Further analysis found that among the five frailty components, pTau was mainly related to weakness (grip strength), exhaustion, and inactivity in frailty. The mechanism may be related to that Tau promotes insulin-induced tyrosine phosphorylation of insulin receptor substrate 1 (IRS-1) and inhibits the activation of phosphatase and tensin homolog (PTEN). Tau deletion leads to an impaired response to insulin caused by altered IRS-1 and PTEN (phosphatase and tensin homologue on chromosome 10) activities [42]. Animal experiments showed that Tau knockout mice exhibited enhanced food intake when fed ad libitum and body weight gain when compared with wild-type littermates in the absence of a change in body weight at weaning. Adiposity was increased in Tau knockout mice, as exemplified by enhanced circulating leptin and adipose tissue weight. Furthermore, Tau deletion was also associated with significant hyperinsulinemia and glucose intolerance [42–44]. Conversely, neuronal accumulation of Tau enhanced insulin responsiveness in transgenic mice, as well as their resistance to a high-fat diet. Even if these mice displayed hypertriglyceridemia and high cholesterol under a high-fat diet, in contrast to wild-type mice, this excess in fat did not convert into an increase in adipose tissue content. Moreover, under a high-fat diet, Tau transgenic mice remained hypoleptinemic and hypoinsulinemic compared to wild-type littermates [45]. This process may contribute to the weight loss observed in individuals [46]. Interestingly, loss of weight and muscle mass are among the hallmarks of frailty. These results suggest that frailty is mainly associated with the Tau-related pathway, especially with active pTau. Regarding the exact mechanism, observations with more samples and more in-depth molecular and cell biology studies are needed.

Growing evidence supports a strong link and overlap between frailty and cognitive impairment [18, 47, 48]. Cross-sectional studies have shown that prefrail and frail individuals aged 50 and older have worse cognitive function than those who are robust [49]. And the participants classified as the most severe degree of frailty exhibited more cognitive domains affected and to a higher degree than participants who were moderately frail and robust [50]. Longitudinal analyses studies in Chinese older adults have further demonstrated that co-existing of frailty and cognitive impairment increases the risk of developing neurocognitive impairment [48]. There is also a research showing that those who were robust but cognitively impaired were more likely to develop pre-frailty/frailty after 4 years compared to those who were robust and cognitively intact at baseline [51]. At the same time, compared to non-frail people, frail people were more than twice as likely to experience cognitive decline. Physical frailty was associated with longitudinal decline in overall cognitive function over two years in the non-demented older adults [52]. The above scientific evidences have shown the bidirectional link between physical frailty and cognitive impairment, which has led to the development of the term "cognitive frailty" in recent years [18, 53]. This suggests an interrelated neuropathology underlying these two constructs [28], so it is plausible to investigate the associations of plasma biomarkers of neurodegeneration with frailty. Here, in our study, after excluding the individuals who were ever diagnosed with AD or other psychoses, pTau was associated with frailty in fully adjusted model, suggesting that pTau was potentially associated with frailty among participants without cognitive impairment or on the early stage of AD. However, the specific biological process behind remains to be clearly defined.

Further studies are required to validate our findings due to several limitations of our study. First, we investigated only a relatively small number of cases. Second, due to the inherent weaknesses of the cross-sectional design, no causal relationships could be inferred from our cross-sectional data, so follow-up studies are needed to establish a causal relationship between pTau and frailty. Third, the participants were mostly community-dwelling older adults, which may limit the generalization of these results to populations with different characteristics. In addition, the correlation between frailty and other cognitive biomarkers was not reflected in this study, probably because our study was limited to plasma and did not include cerebrospinal fluid. Cognition-related biomarkers may also affect frailty through alterations in brain structure and function, which relies on radiological measurements such as MRI and PET and other neuroimaging markers. Future research on frailty should not ignore biomarkers in hematology, cerebrospinal fluid, and other human specimens and should also focus on exploring the role of imaging in them. Despite these limitations, they do not affect the results and trends derived from this study. To our best knowledge, this is the first work to investigate the associations of frailty with blood-based Tau levels, focusing solely on older adults.

In conclusion, the present study found a potential association between pTau and frailty, but the underlying pathophysiological mechanisms remain to be investigated. Future works should monitor the longitudinal trajectory of changes in pTau concentrations and frailty in older adults. A better understanding of the molecular mechanisms behind cognition and frailty will contribute to biomarker research in this area.

## Data Availability

The data that support the findings of this study are available on request from the corresponding author. The data are not publicly available due to privacy or ethical restrictions.
